# From Static to Dynamic: The Convergence of Nanomaterials and 3D/4D Bioprinting for Adaptive Wearable Sports Biosensors

**DOI:** 10.3390/bios16070392

**Published:** 2026-07-20

**Authors:** Haya Akkad, Fatih Ciftci, Esma Ahlatcıoğlu Özerol, Ahmet Akif Kizilkurtlu

**Affiliations:** 1 Department of Bioengineering, Faculty of Chemical and Metallurgical Engineering, Yildiz Technical University, Istanbul 34220, Turkey; hayaakkad5@gmail.com (H.A.); eahlatci@yildiz.edu.tr (E.A.Ö.); 2 Faculty of Engineering, Department of Biomedical Engineering, Fatih Sultan Mehmet Vakıf University, Zeytinburnu, Istanbul 34015, Turkey; fciftci@fsm.edu.tr; 3 BioriginAI Research Group, Department of Biomedical Engineering, Fatih Sultan Mehmet Vakıf University, Zeytinburnu, Istanbul 34015, Turkey; 4 Biomedical Electronic Design Application and Research Center (BETAM), Fatih Sultan Mehmet Vakıf University, Istanbul 34220, Turkey; 5 Faculty of Engineering and Natural Sciences, Department of Biomedical Engineering, Atlas University, Istanbul 34220, Turkey

**Keywords:** 4D printing, stimuli-responsive materials, MXene, self-healing biosensors, microfluidics, sports physiology, shape memory polymers

## Abstract

Wearable biosensors have swiftly progressed from stiff laboratory prototypes to flexible, skin-like systems capable of ongoing physiological monitoring. Yet, the active and mechanically intense nature of athletic performance reveals the limits of static device designs made solely through traditional 3D printing. This review offers a thorough analysis of the shift from custom 3D-printed platforms to adaptive 4D-printed wearable biosensors that include time-sensitive, stimuli-responsive materials. We carefully investigate how nanomaterial-engineered transducers, including carbon nanomaterials, MXenes, and metallic nanostructures, improve electrochemical sensitivity, signal stability, and mechanical durability in sweat-based and electrophysiological sensing. Additionally, we examine the integration of thermoresponsive polymers, moisture-activated hydrogels, shape-memory materials, and self-healing networks that support autonomous control of skin–sensor contact, microfluidic sweat management, and structural stability under high mechanical strain. Case studies focused on sports monitoring demonstrate how these innovations enable multimodal measurement of mechanical, chemical, and molecular biomarkers in real time. Lastly, we address manufacturing scalability, regulatory issues, and translational challenges necessary to move from proof-of-concept to clinical deployment. By combining nanomaterial-driven electrochemical precision with 4D-printed mechanical adaptability, this review maps a path toward adaptive, self-regulating wearable platforms that sustain analytical accuracy even under extreme physiological demands.

## 1. Introduction

Wearable biosensing technologies have become a transformative platform for continuous, non-invasive, real-time monitoring of physiological and biochemical parameters [1]. Advances in flexible electronics, soft materials, and microfabrication have enabled the creation of skin-conformal sensors that detect physical signals, metabolites, and molecular biomarkers directly from biofluids like sweat and interstitial fluid [2]. These systems offer significant promise for personalized medicine, early disease detection, and closed-loop therapeutic interventions. Despite rapid technological advances, the translation of wearable biosensors from lab prototypes to clinically validated and commercially scalable medical devices remains limited. While many sensing methods have shown high analytical sensitivity and selectivity under controlled conditions, real-world use presents complex challenges related to biological variability, mechanical stability, signal reliability, and long-term biocompatibility [2,3,4]. Variations in biofluid composition, environmental exposure, and user-specific physiology can significantly affect sensor performance, making calibration and standardization across populations more challenging [2,4].

One of the major challenges in wearable biosensing is the significant inter- and intra-individual variability observed in biofluids. Factors such as age, sex, fitness level, hydration status, diet, circadian rhythm, medication use, and environmental conditions can influence biomarker concentrations in sweat and other biofluids. For example, sweat rate and electrolyte composition may vary substantially between individuals performing the same physical activity under similar conditions. These variations can introduce measurement bias and reduce the accuracy of universal calibration models [2,4].

Furthermore, environmental factors, including temperature, humidity, and physical exertion, can alter sweat secretion dynamics, thereby affecting analyte transport and sensor response. As a result, calibration strategies developed under controlled laboratory conditions may not always translate reliably to real-world applications [5,6,7]. To address these challenges, recent studies have proposed personalized calibration approaches, machine learning-assisted signal correction, multimodal sensing frameworks, and population-specific reference databases. Standardization efforts are also focusing on harmonized testing protocols, reference materials, and validation procedures to improve comparability across studies and facilitate clinical translation of wearable biosensors [2,4,8,9,10].

Wearable sensors can be systematically classified based on the type of physiological data they collect. This functional grouping usually covers the monitoring of mechanical and electrophysiological signals, biochemical analytes, and molecular biomarkers [11,12]. Such classification offers a structured way to assess sensing mechanisms, device designs, and data analysis methods. It also supports the integration of different sensing methods, helping to develop multimodal monitoring systems that track complex physiological responses during physical activity and training [11,12,13,14,15,16].

This review was developed through a comprehensive literature survey focusing on recent advances in wearable biosensors, nanostructured sensing materials, additive manufacturing technologies, and smart biointerfaces for physiological monitoring. Particular emphasis was placed on publications from 2020 to 2025 to capture the most recent developments in 3D and 4D-printed wearable systems, stimuli-responsive materials, multimodal sensing platforms, and emerging biofluid-monitoring technologies. The selected studies were first organized and summarized according to sensing principles, material platforms, fabrication approaches, and application areas. Subsequently, the collected literature was comparatively analyzed to identify common technological trends, advantages, limitations, translational challenges, and future opportunities in wearable biosensing technologies. Thus, the review was designed not only to summarize representative studies but also to provide a critical assessment of the current state of the field and its future development directions.

### Advanced Wearable Sensing Platforms for Multimodal Physiological Assessment

Recent advances in wearable sensing platforms have shifted the field from single-parameter measurement toward real-time, multimodal physiological assessment. These integrated systems combine mechanical, electrical, and chemical sensing capabilities within a single platform, enabling thorough and dynamic evaluation of physiological states [13]. Multimodal sensing requires interdisciplinary approaches that include sensor fusion, data integration, and high-resolution biosignal processing [9,13]. This capability is especially valuable for monitoring performance, metabolic stress, and neuromuscular adaptation in real time, offering a more comprehensive understanding of human physiology under different physical conditions [4,9,13].

Material-related constraints further limit clinical adoption. Flexible substrates and biofunctional interfaces must simultaneously meet mechanical durability, chemical stability, and sterilization compatibility without sacrificing sensing performance. Additionally, integrating devices into scalable manufacturing processes remains difficult due to variability in microfabrication yields and material heterogeneity [10]. These issues emphasize a persistent gap between proof-of-concept demonstrations and the practicality of industrial production. Regulatory requirements add another layer of complexity. Continuous monitoring devices must satisfy strict safety, biocompatibility, and performance standards, including long-term skin-contact testing, algorithm validation, and verification of clinical outcomes [9]. For multi-analyte platforms and closed-loop systems, regulatory pathways become more complex due to their combined diagnostic and therapeutic functions. Given these challenges, a systematic assessment of technological maturity, performance limitations, and translational barriers is crucial [8,9,10].

## 2. Engineering the Transducer: Nanomaterials for Electrochemical Precision

The analytical performance of wearable electrochemical biosensors is fundamentally governed by the functional materials in the transducer layer and their interactions with complex biological matrices, such as sweat [17]. Non-invasive biofluid sensing imposes strict requirements, including high sensitivity to low analyte concentrations, mechanical stability under repeated deformation, resistance to biofouling, and long-term biochemical stability. Consequently, modern wearable biosensors typically employ multilayer architectures that integrate conductive nanomaterials, flexible substrates, hydrogel biointerfaces, selective biorecognition elements, and microfluidic sampling components [18]. In sweat-based sensing, material selection directly determines key analytical performance metrics, including sensitivity (µA mM^−1^ cm^−2^), limit of detection (LOD), linear dynamic range, response time, mechanical strain tolerance, signal stability, and selectivity coefficients [19]. Across the literature, wearable electrochemical sensors typically achieve sensitivities in the range of 10–10^3^ µA mM^−1^ cm^−2^, depending on the analyte and electrode architecture; detection limits from sub-micromolar to low micromolar; response times below 30 s [18]; and operational stability spanning several days to multiple weeks under intermittent use. Mechanically compliant systems often tolerate 20–100% tensile strain without significant signal degradation, although long-term drift remains a major engineering challenge [20].

To mitigate long-term signal drift, several engineering strategies have been proposed in recent studies on wearable biosensors. Material-based approaches include anti-biofouling coatings, zwitterionic surface modifications, antioxidant stabilization layers, and self-healing conductive networks to preserve electrode integrity and mitigate performance degradation during prolonged operation [21,22,23]. Device-level solutions involve incorporating reference electrodes, redundant sensing channels, and microfluidic architectures to maintain stable analyte transport and minimize environmental fluctuations [21,22,23]. In parallel, data-driven approaches such as adaptive calibration algorithms, machine learning-assisted signal correction, and real-time baseline compensation have emerged as promising methods to improve long-term measurement stability [21,22,23]. The combination of material engineering, device optimization, and computational correction is increasingly viewed as a key strategy for achieving reliable long-term operation in wearable biosensing platforms [21,22,23].

Material engineering must also address the intrinsic complexity of sweat as an analytical medium. Sweat contains low concentrations of many metabolites, exhibits highly variable secretion rates, and displays significant fluctuations in pH and ionic strength [20,24,25]. Additional complications include evaporation, contamination from the skin surface, and strong inter-individual variability in sweat composition [19]. These factors introduce measurement noise, analyte dilution effects, and signal instability. Functional materials are therefore designed to enhance mass transport control, maintain stable electrode–electrolyte interfaces, and resist nonspecific adsorption, thereby preserving measurement accuracy under physiologically dynamic conditions [24,25].

### 2.1. Classification of Materials Used in Wearable Sensors

#### 2.1.1. Carbon-Based Nanomaterials

Carbon nanomaterials remain the most widely implemented transducer materials in wearable electrochemical sensing due to their high electrical conductivity, large electroactive surface area, and structural flexibility [26]. Graphene-based electrodes typically exhibit sensitivities of 50–500 µA mM^−1^ cm^−2^ for metabolites such as glucose and lactate, with detection limits typically in the low micromolar range [27]. Reduced graphene oxide provides enhanced electron transfer kinetics due to restored conjugated networks, while graphene oxide supports biomolecule immobilization through abundant oxygen-containing functional groups. Carbon nanotube networks provide high-aspect-ratio conductive pathways that enable rapid charge transport and mechanical resilience. Their piezoresistive behavior supports simultaneous biochemical and mechanical sensing [28]. CNT-modified electrodes often exhibit response times below 10 s and maintain stable performance under cyclic strain of 30–50%. Porous carbon and carbon black enhance the electroactive surface area and facilitate enzyme loading, thereby improving signal amplification while maintaining low fabrication costs. Carbon materials also contribute to sweat matrix stabilization by improving electrode wetting behavior and enabling mediator-free electron transfer, reducing interference from fluctuating ionic strength. Surface functionalization strategies further improve resistance to protein adsorption and biofouling, which is critical for long-term epidermal operation [26,27,28].

#### 2.1.2. Mxene-Based Two-Dimensional Materials

MXenes have emerged as high-performance electrochemical transducers due to their metallic conductivity, hydrophilic surfaces, and abundant functional groups, which enable strong interfacial coupling with biological molecules [29]. Ti_3_C_2_T_x_-based electrodes frequently demonstrate sensitivities exceeding 500 µA mM^−1^ cm^−2^ and detection limits below 1 µM for ionic and metabolite sensing [30]. Their hydrophilicity promotes rapid electrolyte penetration, stabilizing signal response under varying sweat secretion rates. MXene films also maintain conductivity under large mechanical deformation, with reported strain tolerances of 40–80%, depending on the composite formulation. Strong interfacial adhesion with elastomeric substrates reduces delamination during long-term wear. However, oxidation-induced loss of conductivity remains a known limitation. Surface passivation, polymer encapsulation, and antioxidant stabilization strategies are therefore frequently employed to extend operational stability beyond several days of continuous exposure to aqueous biofluids [29,30,31].

#### 2.1.3. Metal Nanostructures

Metal nanostructures enhance electrochemical reaction kinetics and enable highly efficient electron transfer [32]. Gold nanoparticle-modified electrodes typically increase sensitivity by one to two orders of magnitude compared with bare electrodes and provide stable platforms for thiol-based covalent immobilization of enzymes and antibodies. Platinum nanoparticles are widely used as catalytic amplifiers in enzymatic sensing systems, significantly reducing response time and improving detection limits [32,33]. Silver nanowire networks form stretchable conductive meshes that maintain electrical continuity under strain exceeding 50%, enabling mechanically robust epidermal electrodes. Liquid metals, such as gallium–indium alloys, form deformable, conductive pathways that self-reconfigure under mechanical stress, enabling extreme stretchability. Metal nanostructures also enable mediator-based electrochemical systems that reduce overpotential requirements and improve selectivity in complex ionic environments such as sweat [32,33,34,35,36]. Table 1 summarizes common material classes used in wearable electrochemical biosensors.

#### 2.1.4. Flexible Polymer Substrates and Elastomers

Elastomeric substrates provide the mechanical compliance required for conformal skin attachment [37]. PDMS-based systems typically support strain levels of 30–70% without structural failure, while Ecoflex can exceed 100% elongation. Thermoplastic substrates, such as polyimide, enable the microfabrication of high-resolution electrode patterns compatible with printed electronics [38]. Long-term epidermal integration requires stable skin–sensor electrical impedance. Mechanical mismatch between rigid electrodes and soft tissue can cause impedance drift and motion artifacts. Breathable substrates reduce sweat accumulation and minimize occlusion-induced irritation, while adhesive interface engineering improves signal stability during prolonged wear [37,38,39].

Mechanical mismatch arises because conventional metallic or rigid electronic components have elastic moduli several orders of magnitude higher than those of soft biological tissues. During body movement, stretching, bending, or compression of the skin causes relative displacement between the electrode and the tissue surface [40,41]. This deformation alters the effective contact area and interfacial pressure at the electrode–skin interface, resulting in fluctuations in contact impedance. Consequently, variations in charge-transfer efficiency and ionic conduction can lead to baseline drift, signal instability, and motion-induced artifacts, particularly in electrophysiological measurements such as ECG, EMG, and bioimpedance monitoring [40,41]. To address this issue, flexible substrates, conductive hydrogels, stretchable electrodes, and mechanically compliant interface materials have been developed to better match the mechanical properties of biological tissues and maintain stable electrical contact during prolonged wear [40,41].

#### 2.1.5. Hydrogel-Based Biointerfaces

Hydrogels provide hydrated, mechanically matched interfaces that reduce contact impedance and stabilize electrochemical signals [42,43]. Conductive hydrogel electrodes typically exhibit impedance reductions of 30–70% relative to dry electrodes [43]. Their porous structure enables controlled analyte diffusion and improves signal reproducibility under fluctuating sweat flow conditions. Advanced hydrogel systems incorporate self-healing networks and double-network reinforcement to maintain mechanical integrity under cyclic loading exceeding thousands of deformation cycles. Anti-biofouling modifications such as zwitterionic polymers further enhance long-term signal stability [42,43,44].

Despite the rapid progress of wearable sensing technologies, several limitations continue to restrict their long-term performance and clinical translation. Conventional sensor platforms often suffer from insufficient mechanical adaptability, limited biocompatibility, signal instability under dynamic conditions, and challenges associated with continuous biofluid monitoring. These limitations have stimulated extensive research into advanced functional materials, nanostructured interfaces, and additive manufacturing technologies to improve sensor conformity, durability, and analytical performance. Consequently, the development of smart materials and next-generation fabrication approaches has emerged as a central focus in wearable biosensor research, providing the foundation for the topics discussed in the following sections [45,46].

### 2.2. Biorecognition Elements and Enzyme Immobilization Strategies

Analytical selectivity depends critically on immobilization of biorecognition molecules. Enzyme-based sensing systems require stabilization strategies to prevent denaturation and loss of activity [47]. Common immobilization methods include covalent bonding to functionalized nanomaterials, physical entrapment within polymer matrices, crosslinking networks, and adsorption onto nanostructured scaffolds [47,48].

Redox mediator systems are often integrated to facilitate electron transfer between enzyme active sites and electrodes, improving sensitivity and reducing interference [49]. Enzyme degradation mechanisms include thermal denaturation, proteolytic cleavage, and conformational instability induced by dehydration or ionic fluctuations [48]. Protective polymer coatings and nanostructured supports are therefore used to maintain catalytic activity over extended periods of operation. Molecularly imprinted polymers provide synthetic recognition sites with high chemical stability, offering alternatives to fragile biological receptors in harsh environments [47,48,49].

### 2.3. Microfluidic Sweat Handling Systems

Microfluidic architectures regulate sweat transport, prevent evaporation-induced concentration changes, and ensure consistent analyte exposure [19]. Channel geometries control residence time, while hydrophilic coatings enhance passive fluid uptake. Paper-based systems enable low-cost capillary transport, whereas elastomeric microchannels support dynamic flow regulation [19]. Controlled fluid delivery reduces signal variability caused by fluctuating sweat secretion rates and improves reproducibility across individuals [1].

#### Osmotic, Porous Hydrogel, and Janus Textile Interfaces for Passive Biofluid Harvesting

Recent advances in wearable biosensing have expanded beyond conventional microfluidic sweat collection systems toward passive biofluid-harvesting interfaces capable of continuous operation under low sweat rates and resting conditions. Among these emerging approaches, osmotic gels, porous conductive hydrogels, and Janus textile architectures have attracted considerable attention for their ability to enhance biofluid extraction, transport, and sensing reliability without external pumping systems or active stimulation [50,51,52,53,54,55,56].

Osmotic gel-based platforms utilize concentration gradients to generate osmotic pressure, enabling the continuous extraction and transport of sweat or interstitial fluid (ISF) toward sensing interfaces. Unlike traditional sweat sensors that rely on active perspiration, osmotic systems can facilitate biofluid collection even with low sweat production, thereby extending wearable monitoring beyond exercise-induced sweating. Recent studies have demonstrated that osmotic materials can enhance sampling consistency, reduce fluid stagnation, and improve temporal continuity in continuous monitoring applications [50,51,52,53,54,55,56].

Porous conductive hydrogels represent another important class of passive biofluid interfaces. Their highly interconnected porous networks simultaneously function as biofluid reservoirs, conductive pathways, and mechanically compliant skin-contact layers. The porous architecture facilitates rapid sweat uptake and transport while minimizing evaporation-induced concentration fluctuations. In addition, the hydrated nature of conductive hydrogels reduces skin–electrode impedance and improves signal stability during long-term operation. Such materials have demonstrated considerable potential for continuous sweat monitoring by maintaining a stable microenvironment at the sensor–skin interface and improving analytical reproducibility [50,51,52,53,54,55,56].

Janus textile systems have recently emerged as promising solutions for directional biofluid management. These materials combine hydrophilic and hydrophobic layers with asymmetric wettability, enabling unidirectional transport of sweat away from the skin toward sensing regions. This directional fluid transport minimizes backflow, reduces local sweat accumulation, and improves the freshness of collected samples. Furthermore, Janus architectures can enhance sweat harvesting efficiency while maintaining wearer comfort and reducing skin irritation during prolonged monitoring [50,51,52,53,54,55,56].

Collectively, osmotic gels, porous conductive hydrogels, and Janus textile interfaces represent a new generation of smart biofluid-management materials that complement microfluidic sensing systems. Their integration with nanostructured transducers and adaptive wearable platforms may significantly improve sampling reliability, analyte transport efficiency, and long-term sensing performance in future wearable biosensors [50,51,52,53,54,55,56].

### 2.4. Smart and Adaptive Materials

Self-healing conductors restore electrical continuity after mechanical damage, extending operational lifetime and improving device durability under repeated deformation. Shape-memory polymers enable adaptive device conformability through programmable and reversible shape transformations triggered by thermal or environmental stimuli. Similarly, stimuli-responsive hydrogels can dynamically regulate their swelling behavior, permeability, and electrical conductivity in response to changes in temperature, moisture, pH, or ionic composition. These adaptive material responses help reduce mechanical fatigue, preserve stable sensor–skin contact, and maintain sensing reliability during long-term operation in wearable applications [57].

### 2.5. Manufacturing Scalability and Translational Fabrication

Scalable manufacturing is essential for clinical translation. Screen printing and inkjet printing enable low-cost mass production of electrode arrays. Roll-to-roll fabrication supports continuous large-area sensor manufacturing. Laser patterning enables rapid prototyping of microelectrode geometries, while transfer printing allows integration of high-performance materials onto soft substrates. The manufacturing strategy directly influences device reproducibility, cost, and the feasibility of regulatory approval [58,59,60].

### 2.6. Regulatory and Clinical Translation Framework

Clinical deployment of wearable biosensors requires compliance with medical device regulatory standards. Device classification determines the required validation pathways, including analytical performance testing and human-subject trials. Biocompatibility evaluation follows ISO 10993 standards [61], while electrical safety is governed by IEC guidelines. Clinical validation must demonstrate accuracy under real-use conditions, long-term stability, and reproducibility across populations. Regulatory constraints, therefore, impose quantitative performance thresholds and standardization requirements that must be considered during early-stage material and device design [8,62].

**Figure 1:**
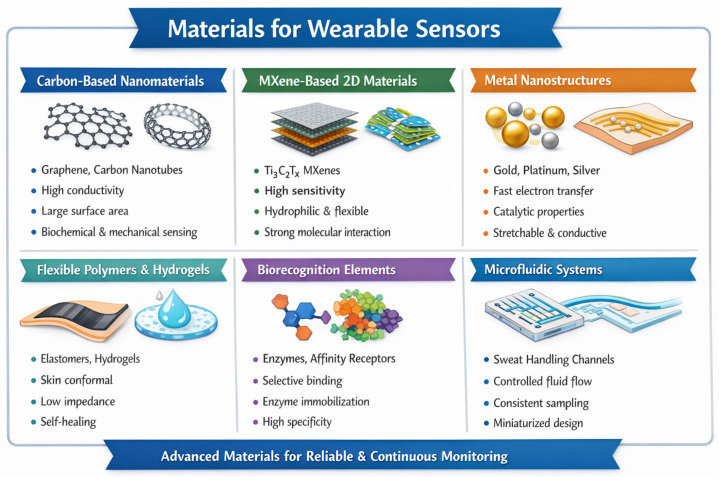
Overview of key material classes used in wearable sensor platforms for continuous physiological monitoring. The schematic highlights major functional components, including carbon-based nanomaterials (graphene and carbon nanotubes) for high electrical conductivity and large surface area, MXene-based two-dimensional materials for high sensitivity and strong molecular interactions, and metal nanostructures (gold, platinum, and silver) for efficient electron transfer and catalytic activity. Image generated by AI using ChatGPT (GPT-5, OpenAI, San Francisco, CA, USA).

Modern wearable biosensors rely on the synergistic integration of conductive nanomaterials, compliant mechanical supports, selective recognition chemistries, and controlled biofluid-handling systems. This materials-driven engineering framework enables reliable, continuous, and clinically meaningful physiological monitoring, bridging the gap between laboratory sensing technologies and real-world biomedical applications [63]. Figure 1 highlights major material classes used in wearable sensor systems for continuous physiological monitoring.

**Table 1:** Comparative performance characteristics of material classes frequently employed in wearable electrochemical biosensors.

Material Class	Typical Sensitivity (µA mM^−1^ cm^−2^)	Limit of Detection	Linear Range	Response Time	Mechanical Strain Tolerance	Operational Stability	Relative Advantages	Relative Limitations	References
Graphene/rGO	50–500	0.1–10 µM	µM–mM	3–20 s	20–40%	7–30 days	Excellent sensitivity, rapid electron transfer, high flexibility, suitable for multiplex sensing	Complex large-scale fabrication, performance variability between batches	[26,64,65]
Carbon Nanotubes (CNTs)	100–800	0.05–5 µM	µM–mM	<10 s	30–60%	2–4 weeks	High conductivity, strong electrocatalytic activity, excellent strain sensing capability	Aggregation tendency, potential biocompatibility concerns	[66,67]
Porous Carbon/Carbon Black	20–200	1–50 µM	µM–high mM	5–30 s	substrate dependent	weeks	Cost-effective, scalable production, large electroactive surface area	Lower sensitivity compared with MXenes and CNTs	[68]
MXene (Ti_3_C_2_Tx)	300–1500	<1 µM	nM–mM	1–10 s	40–80%	5–14 days (oxidation limited)	Highest sensitivity, ultrafast response, excellent conductivity and hydrophilicity	Oxidation susceptibility and limited long-term stability	[69,70,71]
Gold Nanoparticles	Signal amplification dependent	down to nM (enzyme systems)	wide	fast kinetics	substrate dependent	weeks	Outstanding bioconjugation capability, excellent signal amplification	High material cost and limited mechanical functionality	[33]
Silver Nanowires	Used as a conductor (not primary sensing)	—	—	—	50–100%	weeks	Highly stretchable and transparent conductive networks	Mainly conductive elements rather than primary sensing materials	[32,35]
Platinum Nanoparticles	Strong catalytic gain	sub-µM	wide	<5 s	substrate dependent	weeks	Superior catalytic performance and fast electron-transfer kinetics	High cost and limited flexibility without composite integration	[32,34]
Conductive Hydrogels	10–150 (interface mediated)	µM	µM–mM	5–30 s	50–200%	days–weeks, hydration dependent	Excellent skin conformity, low interfacial impedance, high stretchability	Dehydration and mechanical degradation during prolonged operation	[44,72]
Enzyme-Functionalized Electrodes	100–1000+ (target dependent)	nM–µM	narrow–moderate	seconds	matrix dependent	3–14 days (activity decay)	Highest molecular selectivity and target specificity	Enzyme denaturation and limited operational lifetime	[73]
Microfluidic Sweat Systems	Indirect influence	Improves effective LOD	Stabilizes range	Improves response reproducibility	flexible	long-term	Improved sampling reliability, reduced evaporation, enhanced reproducibility	Increased fabrication complexity and integration challenges	[17,74]

Flexible polymers and hydrogels are shown as skin-conformal substrates that provide mechanical compliance, low impedance, and structural stability. Biorecognition elements, such as enzymes and affinity receptors, enable selective and specific target detection through molecular binding and immobilization strategies. Integrated microfluidic systems are also illustrated for controlled handling of biofluids, consistent sampling, and device miniaturization. Together, these material systems form the foundation for reliable, flexible, and high-performance wearable biosensing technologies [61].

## 3. 3D Printing: Customization and Microfluidic Management

### 3.1. Anatomical Conformity via 3D Scanning

Precise anatomical conformity is essential for signal stability, comfort, and durability in wearable biosensing systems. Traditional wearable devices are usually designed with standard shapes that overlook individual differences in body shape, tissue softness, or localized movements. Consequently, poor skin contact, uneven pressure, and mechanical stress in specific areas can reduce sensor effectiveness and user comfort, especially during long or intense physical activities [75,76].

Three-dimensional scanning technologies allow for the creation of high-resolution digital models of anatomical surfaces, forming the foundation for fully customized sensor design. Imaging methods such as optical surface scanning, structured light scanning, and medical imaging techniques like MRI or CT can capture subject-specific geometry with sub-millimeter accuracy. These anatomical datasets are then processed using computer-aided design (CAD) software to develop device structures that precisely fit the target body region. Additive manufacturing techniques, including material extrusion, stereolithography, and multi-material printing, are used to convert the digital model into a physical device [75,76,77].

This workflow enables the creation of customized wearable platforms that maintain consistent mechanical contact with biological tissues, thereby reducing motion-related signal artifacts. In sports and performance monitoring, anatomical customization is especially important for areas exposed to high mechanical stress or repetitive deformation. For instance, personalized mouthguards with electrochemical sensing elements can be made to monitor salivary biomarkers without disrupting breathing, speech, or occlusion mechanics. Likewise, custom-fit insoles with pressure sensors and sweat-monitoring features can be designed to match the plantar surface, ensuring stable measurements during running and other high-impact activities while maintaining natural gait dynamics. Anatomically tailored structures enhance not only mechanical stability but also measurement consistency, as steady contact pressure and minimized micro-movements lower inter-session variability. From an applied perspective, personalized manufacturing improves ergonomics, increases device retention during vigorous activity, and reduces the chances of skin irritation or pressure-related tissue damage [75,76,77].

### 3.2. Microfluidic Architectures for Controlled Sweat Routing

Accurate biochemical sensing in wearable platforms depends on controlled sampling and transport of biofluids from the skin surface to the sensing interface. Sweat secretion is naturally dynamic, spatially variable, and responsive to environmental and physiological conditions. Without proper fluid management, the analyte concentration at the sensor interface can be affected by evaporation, dilution, contamination, or mixing of sweat over time [75].

Three-dimensional printing enables the precise fabrication of microfluidic structures that control sweat collection, transport, and delivery with high spatial resolution. Capillary-driven microchannels are often used to enable passive fluid movement without external pumps. These channels are carefully designed with specific cross-sectional shapes, surface energies, and hydraulic resistances to manage flow speed and direction. Hydrophilic surface treatments further enhance capillary action, ensuring rapid and consistent fluid uptake directly from eccrine sweat glands [77].

One of the main challenges in sweat sensing is the buildup and mixing of previously secreted sweat with newly produced fluid. This timing mixing changes analyte concentration profiles and causes measurement delays, reducing the accuracy of real-time monitoring. Microfluidic designs address this problem by incorporating directional flow paths, flow resistors, and fluid-isolation chambers that separate sequential sweat samples. Some designs use one-way transport structures or sequential reservoirs to maintain temporal resolution, ensuring only freshly secreted sweat reaches the sensing electrode. Additional microfluidic features may include evaporation-control layers, bubble traps, and flow-rate calibration structures to stabilize analyte concentration under varying sweating conditions. By controlling residence time and reducing diffusion-driven mixing, these systems help maintain the timing accuracy of biochemical measurements [75,76].

**Figure 2:**
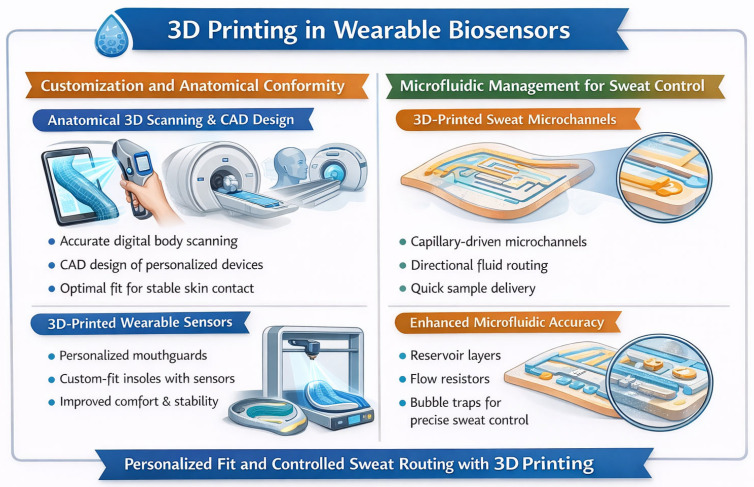
Schematic illustration of how 3D printing supports the development of wearable biosensors, focusing on device customization and microfluidic sweat management. The figure illustrates how combining anatomical 3D scanning with computer-aided design (CAD) enables the creation of customized wearable devices that conform more closely to the skin and maintain stable contact. Image generated by AI using ChatGPT (GPT-5, OpenAI, San Francisco, CA, USA).

By integrating anatomically conforming structures with precisely designed microfluidic networks, 3D printing enables wearable biosensors to achieve both stable mechanical attachment and controlled biochemical sampling. This combination of structural and fluidic precision enables accurate real-time monitoring even in dynamic physiological conditions, especially in high-performance sports settings where movement and sweat levels constantly change [63,75,76,77]. Figure 2 illustrates how 3D printing supports the development of wearable biosensors.

Examples of 3D-printed wearable platforms, such as personalized mouthguards and sensor-integrated insoles, are provided to highlight enhanced comfort and stability. Additionally, the integration of 3D-printed microfluidic architectures demonstrates precise sweat control, including capillary-driven microchannels, directional fluid routing, and rapid sample transport. Advanced microfluidic features—such as reservoir layers, flow resistors, and bubble traps—are shown to enhance sampling accuracy and regulation. Overall, the figure demonstrates how additive manufacturing enables personalized device production and controlled transport of biofluids, supporting reliable wearable biosensing [63,75,76,77].

## 4. 4D Printing: Smart Materials for Autonomous Adaptation

Four-dimensional printing introduces programmable material behavior into wearable biosensing platforms, enabling structures that dynamically respond to physiological and environmental stimuli. Unlike passive flexible systems, these materials exhibit time-dependent transformations in shape, mechanical properties, or fluid regulation without external control circuitry. This adaptive functionality improves signal stability, preserves biointerface integrity, and boosts operational robustness in physiologically dynamic environments such as athletic performance monitoring [78].

### 4.1. Thermal Actuation for Robust Skin–Sensor Coupling

Thermal-responsive materials enable wearable systems to actively control skin contact based on changes in body temperature. During physical activity, increased sweating and higher skin temperatures reduce interfacial friction, leading to sensor displacement. Passive elastic structures cannot counteract these thermally induced lubrication effects, leading to a gradual deterioration of the signal [78].

Poly(N-isopropylacrylamide) (PNIPAM) is one of the most widely used thermoresponsive polymers in adaptive biointerfaces. PNIPAM has a lower critical solution temperature (LCST) near body skin temperature (~32–34 °C). Below this temperature, the polymer stays hydrated and swollen; above it, the polymer quickly loses water and shrinks. This reversible phase change enables thermally triggered tightening of sensor assemblies during exercise-induced temperature increases [79].

Shape memory polymers (SMPs) offer an alternative method for thermal actuation. These materials can be programmed into a temporary shape and then recover a predefined permanent form when exposed to a specific temperature. In wearable systems, SMP-based structural elements can be designed to increase curvature or compressive force at higher temperatures, helping to keep stable epidermal contact under varying thermal conditions [80].

Thermally adaptive wearable bands or support structures made with PNIPAM or SMP components can therefore counteract sweat-induced lubrication by actively increasing conformal pressure as skin temperature rises. This helps keep electrical impedance consistent at the skin–sensor interface, minimizes motion artifacts, and maintains signal continuity during prolonged physical activity [78,79,80,81,82].

### 4.2. Moisture-Responsive Microvalves for Autonomous Fluid Regulation

Moisture-responsive materials enable passive, self-regulating microfluidic control in sweat sampling systems. Hydrogels, made of crosslinked hydrophilic polymer networks, expand volumetrically when they absorb water. This swelling can be precisely adjusted by modifying crosslink density, polymer composition, and environmental sensitivity [80,81].

In wearable sweat sensing platforms, hydrogel-based structures can serve as self-regulating microvalves within microfluidic systems. When exposed to sweat, the hydrogel absorbs fluid and expands, gradually blocking the microchannel that carries sweat. Once a set fluid volume is reached, the channel swells fully, effectively halting further flow through that pathway [47,49,76].

This mechanism allows passive sequential sampling without electronic control. Once a microchannel is sealed, newly secreted sweat is diverted into an alternative channel or reservoir, maintaining separation between sampling intervals. This sequential compartmentalization prevents mixing of earlier and later sweat samples, thereby enhancing the temporal resolution of biochemical monitoring [83].

Moisture-responsive stop-flow regulation stabilizes measurement conditions by controlling fluid residence time and reducing evaporative loss. Since hydrogel swelling is reversible through controlled dehydration or mechanical relaxation, systems can be designed for either repeatable or single-use sampling, based on application needs. By integrating hydrogel-based microvalves directly into 3D- or 4D-printed microfluidic architectures, wearable devices enable autonomous fluid routing that adapts to perspiration dynamics, thereby removing the need for external pumping or active valve control [78,83,84].

### 4.3. Self-Healing Structural and Electrical Integrity in High-Impact Environments

Wearable biosensors used in contact sports endure repeated mechanical stress, including compression, bending, abrasion, and impact. These forces can cause microcracks in conductive pathways, polymer matrices, or interfacial layers, leading to gradual electrical failure and loss of sensing function [85].

Self-healing polymers use reversible or dynamic bonds that enable they can repair themselves after damage. Dynamic covalent bonds, such as disulfide exchange, along with non-covalent interactions, including hydrogen bonds, ionic bonds, and supramolecular assemblies, enable polymer networks to reorganize and heal after breaking. When mechanical stress damages the network, molecular motion helps reform bonds across the damaged areas, restoring the material’s strength [85].

**Figure 3:**
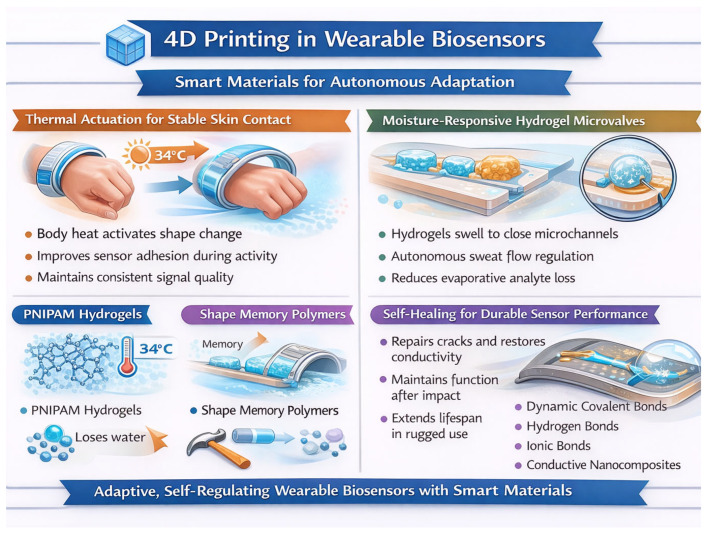
A conceptual illustration of four-dimensional (4D) printing strategies employed in wearable biosensors highlights the utilization of intelligent materials that enable autonomous structural and functional adaptation over time. Image generated by AI using ChatGPT (GPT-5, OpenAI, San Francisco, CA, USA).

In conductive composites, self-healing polymer matrices can restore percolation networks of conductive fillers such as carbon nanotubes, graphene, or metallic particles. This allows electrical conductivity to recover after microstructural disruptions. Healing can occur under ambient conditions or be sped up by mild thermal activation, depending on the material’s chemistry. For wearable sensors used in high-impact activities like rugby, martial arts, or combat training, self-healing functionality extends their operational life and maintains measurement reliability under repeated mechanical stress. Autonomous repair reduces maintenance needs, decreases performance decline over time, and improves device resilience in unpredictable mechanical environments [85,86]. Figure 3 illustrates four-dimensional (4D) printing strategies employed in wearable biosensors.

Figure 3 exemplifies thermally responsive actuation mechanisms designed to improve skin conformity and signal stability through shape modification induced by body heat, as well as moisture-responsive hydrogel microvalves that regulate sweat flow and minimize analyte loss. Representative smart material systems, including PNIPAM-based hydrogels and shape-memory polymers, are depicted for temperature-triggered structural transformations and reversible deformation. Furthermore, self-healing material designs based on dynamic covalent, hydrogen, and ionic bonds, as well as conductive nanocomposites, have been shown to restore electrical conductivity and maintain sensor performance following mechanical damage. In conclusion, the illustration underscores how 4D printing enables the development of adaptive, self-regulating wearable biosensors with enhanced durability, reliability, and long-term operational stability [11,17,85,86,87,88].

## 5. Application-Specific Case Studies

### 5.1. Wearable Sensors That Measure Physical and Electrophysiological Parameters

Wearable sensor technologies are increasingly integral to sports applications, with a focus on performance optimization, physiological load monitoring, and injury risk mitigation. Systems that continuously measure physical, mechanical, and electrophysiological parameters in real time enable the assessment of athlete performance in the field, overcoming the constraints of laboratory environments [89]. In this regard, parameters such as sweat rate and electrolyte concentration are vital for evaluating hydration status, while strain, pressure, and EMG signals offer direct insights into muscle activation, joint mobility, and mechanical load distribution. The studies outlined in this section employ advanced materials and manufacturing techniques to measure parameters that are significant to sports science.

Hashimoto et al. developed an innovative, potentially wearable sensor capable of continuously monitoring both the local sweat rate and sweat electrolyte concentration. The device incorporates a microfluidic system with a short pathway that directs sweat from the skin to a confined chamber, where it forms a measurable droplet. The sweat rate is determined by the time elapsed until a droplet appears and its volume, while an integrated electrical sensor measures the sodium chloride concentration in each droplet. The sensor demonstration verified that this newly developed device can precisely record artificial sweat flow rates and sodium chloride concentrations within ranges characteristic of human sweating, with an accuracy of ±10%. This level of precision is comparable to that of commercially available sweat rate meters and sweat ion sensors. Moreover, it provides a novel perspective for developing wearable sensors for continuous monitoring of sweat rate and electrolyte concentration, with prospective applications in healthcare devices [83].

İslam et al. introduced an extensive wearable platform comprising a fully printed, flexible sensor patch, readout electronics, and a mobile application designed for ongoing, real-time monitoring of perspiration rate. Employing direct 3D printing and scalable microfluidic fabrication, a sensor patch with an area of 700 mm^2^ and a mass of 380 mg was produced. The microfluidic channels are 850 μm wide and 164 μm thick, incorporating serpentine electrodes that measure perspiration rate via capacitance. The custom readout electronics detect capacitance variations to precisely quantify sweat rate, with a sensitivity of 0.01 μL/min. The sensor’s performance was validated by comparing it with analytical models, simulations, and in vivo trials using commercial sensors. This cost-efficient, flexible, and fully integrated sweat-sensing system holds considerable promise for applications in precision health [90].

Zhang et al. developed a three-dimensional porous Ti_3_C_2_T_x_ MXene/poly(3,4-ethylenedioxythiophene) polystyrene sulfonate (PEDOT: PSS) composite aerogel (MPCA) featuring controllable patterning via a Cu-assisted electrogelation technique. The resulting composite aerogel is suitable for assembly into pressure sensors for wearable physical monitoring and into high-resolution sensor microarrays for robotic tactile sensing. The multiple interactions between MXene and PEDOT: PSS confer a stable 3D conductive network on MPCA, thereby enhancing both its mechanical flexibility and piezoresistive properties. Consequently, the fabricated pressure sensor exhibits high sensitivity, rapid response time, and excellent stability, rendering it suitable for wearable physical monitoring applications. Furthermore, owing to the electrogelation process’s controllable patterning capability, a high-resolution pressure sensor microarray was successfully fabricated as an artificial tactile interface that can be affixed to a robotic fingertip to directly detect tactile stimuli from human fingers and identify Braille characters, akin to human tactile perception. The proposed MPCA, characterized by superior overall properties, particularly its highly sensitive sensing performance and controllable patterning capability, demonstrates significant advantages and considerable potential for utilization in wearable electronic devices [91].

Lu et al. developed a highly stretchable, sensitive, and multifunctional flexible strain sensor based on MXene (Ti_3_C_2_T_x_)-composited poly(vinyl alcohol)/polyvinylpyrrolidone double-network hydrogels. The evenly dispersed hydrophilic MXene nanosheets formed a 3D conductive network within the hydrogel, providing the flexible sensor with high sensitivity. The strong interaction between the double-network hydrogel matrix and MXene significantly enhanced the hydrogels’ mechanical properties. These nanocomposite hydrogels demonstrated excellent tensile performance (2400%), toughness, and resilience. Notably, the flexible pressure sensor exhibited ultrahigh sensitivity and a wide response range, quick response time, and a low detection limit. Additionally, hydrogel-based flexible sensors, which combine high sensitivity and durability, can monitor full-range human motion and be assembled into aligned devices to detect subtle pressures, offering great potential in facial expression and speech recognition, handwriting verification, health diagnostics, and wearable electronics [92].

Lee et al. introduced a fabric-based lamina-emergent MXene electrode, a lightweight, flexible wearable device that morphs shape and offers an innovative way to sense biosignals. It leverages MXene’s high electrical conductivity and low skin-to-electrode contact impedance, inspired by Nesler’s pneumatic interference actuator design, to ensure stable contact and reliable biosignal detection across various conditions. Extensive research examined key design factors, including the width and number of semicircular legs, the radius of the anchoring frame, and pneumatic pressure, to suit a broad range of applications. Additionally, a real-time wireless electrophysiological monitoring system was developed, achieving signal-to-noise ratios and accuracy comparable to those of commercial bioelectrodes. This system excels at recognizing hand gestures using a convolutional neural network and introduces a shape-morphing electrode that delivers dependable, high-performance biosignal sensing for dynamic users [93].

Garg et al. presented, validated, and demonstrated the broad clinical applicability of dry, wearable MXene HDsEMG arrays (MXtrodes) fabricated via safe, scalable liquid-phase processing of Ti_3_C_2_T_x_. Their fabrication scheme allows easy customization of the array geometry to match the subject’s anatomy, while the gel-free, minimal skin preparation enhances usability and comfort. The low impedance and high conductivity of the MXtrode arrays enable the detection of EMG activity in large muscle groups with higher quality and spatial resolution than current state-of-the-art wireless EMG sensors, even in realistic clinical scenarios. To demonstrate the clinical applicability of MXtrodes in neuromuscular diagnostics and rehabilitation, the presentation included simultaneous high-density surface electromyography (HDsEMG) and biomechanical mapping of muscle groups across the entire calf during various tasks, ranging from controlled contractions to ambulation. Moreover, the integration of HDsEMG data acquired with MXtrodes into a machine learning framework was demonstrated, enabling precise prediction of human gait phases. These findings highlight the advantages and translational potential of MXene-based wearable bioelectronics for investigating neuromuscular function and disease, as well as for precision rehabilitation [94].

Zhang et al. proposed an adaptive multifunctional strain sensor (AMSS) 4D printing strategy based on the shape-memory properties of polylactic acid (PLA), a bioslit structural sensing unit, and a bidirectional deformation design for a bilayer structure. The obtained AMSS demonstrated excellent sensitivity to strain, mechanical, and temperature stimuli. In particular, thanks to the 3D-printed slit structure and phase-transition properties of the PLA printing layer, the AMSS macro-microstructure can be precisely tuned, and its sensing performance is programmable. The built-in structural design-induced macro-deformation enables AMSS to adaptively fit human joint surfaces to recognize full-range human motion. In addition, the close correlation between strain sensing during AMSS shape transformation enables self-sensing of position and strain in AMSS. Furthermore, by integrating and separating resistive signals, we can detect temperature and mechanical stimuli. Finally, they integrated the wireless sensing module into the AMSS to improve the sensor’s portability and wearability [95].

Deng et al. introduced a 4D printing method to create multistable shape-morphing structures that can be precisely controlled by applied strains. The structures are printed using a two-nozzle 3D printer that spatially distributes phase-change wax microparticles (MPs) within the elastomer matrix. These wax MPs can retain residual strain after the prestrained elastomer composite relaxes due to the solid–liquid phase change. Thanks to the high design flexibility of 3D printing, the spatial distribution of the wax MPs can be programmed, resulting in an anisotropic stress field within the elastomer composite. This causes out-of-plane deformations such as curling, folding, and buckling. These deformations are multistable and can be reprogrammed because of the reversible phase change of the wax MPs. Furthermore, characteristics of the deformations, such as curvatures and folding angles, are linearly related to the applied strains, indicating that these deformations are quantitatively controllable. Finally, the study demonstrates applications of the strain-tailored, multistable shape-morphing 3D structures in assembling 3D electronics and adaptive wearable sensors [96].

Cheng et al. introduced a materials programming method for designing self-shaping material systems produced by 4D printing, inspired by biological models. They developed computational design techniques for 4D printing of biologically inspired behaviors involving complex mechanisms. To emulate the anisotropic arrangement of mobile plant structures, material systems are tailored at the mesoscale using extrusion-based 3D printing. The approach is demonstrated by applying the force-generating principle of a climbing plant (*Dioscorea bulbifera*) to a self-tightening splint. *D. bulbifera* exerts compressive force on its support to maintain stability against gravity by stretching the stem helix. The functional strategies of *D. bulbifera* are abstracted and transferred to custom 4D-printed material systems. The compressive forces of these biologically inspired movement mechanisms are then evaluated. Ultimately, the self-tightening function is prototyped on a wrist-forearm splint, a common orthotic device for alignment. This approach enables the application of innovative biomimetic design strategies to 4D-printed motion mechanisms, expanding the possibilities for new adaptive designs in wearable assistive technologies and beyond [97].

**Figure 4:**
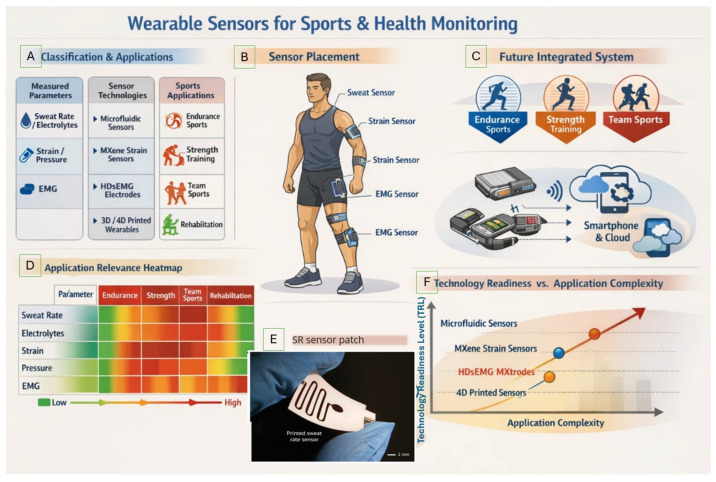
Overview of wearable sensor systems for sports and health monitoring. (**A**) Classification of MXene-based strain sensors, high-density surface EMG electrodes, and 3D/4D-printed wearable platforms, mapped against target sports applications. (**B**) Schematic representation of sensor placement on various body regions, illustrating localized physiological and biomechanical monitoring. (**C**) Architecture of integrated digital health systems in which wearable devices wirelessly transmit data to smartphones and cloud platforms for future automated analysis. (**D**) Qualitative heatmap developed by the authors based on the comparative analysis of the studies summarized in Table 2 [89,90,91,92,93,94,95,96,97,98], illustrating the relative relevance of different physiological parameters across endurance sports, strength training, team sports, and rehabilitation applications. (**E**) Optical image of a flexible, printed sweat rate (SR) sensor patch [90]. (**F**) Based on the literature reviewed, a qualitative comparison of wearable sensor technologies according to their relative maturity and application complexity is presented. The diagram is designed as a conceptual visualization [89,90,91,92,93,94,95,96,97,98]. Image generated by AI using ChatGPT (GPT-5, OpenAI, San Francisco, CA, USA).

Drisscroll et al. introduced MXtrodes, a novel class of soft, high-resolution, large-scale bioelectronic interfaces enabled by Ti_3_C_2_ MXene and scalable solution processing. They demonstrated that the electrochemical properties of MXtrodes surpass those of conventional materials and do not necessitate conductive gels when employed in epidermal electronics. Moreover, we corroborate the efficacy of MXtrodes across applications, ranging from mapping extensive neuromuscular networks in humans to delivering cortical microstimulation in small-animal models. Lastly, they established that MXtrodes are compatible with standard clinical neuroimaging modalities [98]. Figure 4 highlights key physiological and biomechanical parameters measured by wearable sensor systems used for sports and health monitoring.

**Table 2:** Summary of key studies demonstrating the use of wearable sensing technologies in sports performance monitoring and physiological evaluation.

Study	Measured Parameter	Sensor Type	Sports-Related Application	Relative Advantages	Current Limitations	References
Hashimoto et al.	Sweat rate, NaCl	Microfluidic + electrochemical	Endurance sports, hydration monitoring	Quantitative sweat analysis with continuous monitoring capability; suitable for hydration assessment	Requires reliable sweat generation and microfluidic integration	[83]
Islam et al.	Sweat rate	Printed microfluidic capacitive sensor	Long-duration training monitoring	Low-cost, scalable, and wearable-friendly fabrication	Limited biomarker diversity and lower analytical complexity	[90]
Zhang (MPCA)	Pressure	MXene/PEDOT: PSS aerogel	Foot pressure, force distribution	High-pressure sensitivity and robust mechanical performance	Primarily focused on mechanical sensing rather than biochemical monitoring	[91]
Lu et al.	Strain, pressure	MXene-based hydrogel	Joint motion, muscle deformation	Exceptional stretchability and mechanical adaptability	Long-term durability and environmental stability require further validation	[92]
Lee et al.	EMG	Textile-integrated MXene electrode	Hand–arm sports, gesture analysis	Comfortable textile integration and stable EMG acquisition during movement	Signal quality may be influenced by textile degradation and motion artifacts	[93]
Garg et al.	HDsEMG	MXene electrode arrays (MXtrodes)	Gait analysis, muscle fatigue detection	High-density electrophysiological mapping with superior spatial resolution	Increased system complexity and data-processing requirements	[94]
Zhang (AMSS)	Strain, temperature	4D printed PLA sensor	Full-range joint motion tracking	Adaptive sensing through programmable shape transformation	Early-stage technology with limited real-world validation	[95]
Deng et al.	Strain-induced deformation	4D printed elastomer composite	Adaptive wearable sensor platforms	Reconfigurable sensor architecture and multistable behavior	Manufacturing complexity and limited commercialization readiness	[96]
Cheng et al.	Mechanical adaptation	Bio-inspired 4D structures	Sports orthoses and support systems	Biomimetic adaptation and potential for personalized support systems	Mostly proof-of-concept studies with limited quantitative validation	[97]
Driscoll et al.	EMG, stimulation	MXene bioelectronic interface	Neuromuscular monitoring in athletes	High-quality electrophysiological recording without conductive gels	Translation to large-scale wearable deployment remains under investigation	[98]

### 5.2. Wearable Sensors That Measure Chemical Parameters

Ji et al. introduced a “Screen + Wax” printing technique to fabricate these components and develop a “Lego Bricks”-type wearable sweat sensor for monitoring sweat Na^+^ and K^+^. Flexible electrode arrays and paper-based microfluidic layers, serving as modular elements, were produced on polyethylene terephthalate and paper substrates through screen printing and wax printing methods, respectively. Gold nanoparticles and Na^+^/K^+^-selective membranes were applied to the electrode surfaces via electrodeposition and drop-coating. This study emphasizes the exceptional performance of the “Lego Bricks” type wearable sweat sensor in assessing Na^+^ and K^+^ imbalances in sweat from various body regions during exercise and, notably, in tracking physical activity during extended exercise sessions under different interventions. Moreover, the developed “Lego Bricks” wearable ion-electrochemical sensor demonstrates high-throughput sample analysis capabilities with superior performance and cost-efficiency, thereby enabling significant integration of sweat monitoring into physical activity management and providing a valuable tool for intelligent health monitoring [99].

Kim et al. adapted 3D printing technology to develop an innovative, multiplex, cost-effective, and mechanically adaptable, all-inclusive integrated wearable (AIIW) patch. This device incorporates 3D-printed flexible sensors and flexible wearable microfluidic sample-handling (WMFSH) units, which are assembled within a few hours. The AIIW patch has been comprehensively characterized, and its utility for noninvasive, continuous health monitoring has been demonstrated through simultaneous ex situ and in situ measurements of multiple electrolyte levels in sweat. This research is regarded as a significant advancement toward facilitating personalized health monitoring by utilizing 3D printing technology to efficiently and affordably create customized, integrated, and flexible wearable biosensing platforms for individual health assessment parameters [100].

**Figure 5:**
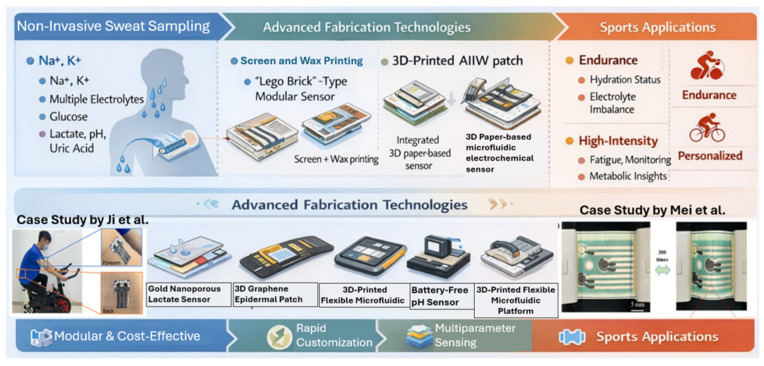
A schematic depiction of wearable sensor technologies designed for non-invasive monitoring of chemical parameters in sports via sweat analysis. The illustration outlines key analytes in sweat, including electrolytes (Na+, K+), glucose, lactate, pH, and uric acid, alongside advanced fabrication methods such as screen printing, wax printing, modular sensor architectures, and three-dimensional-printed microfluidic platforms. Image generated by AI using ChatGPT (GPT-5, OpenAI, San Francisco, CA, USA).

As shown in Figure 5, Mei et al. introduced an integrated 3D paper-based microfluidic electrochemical sensor for in situ sweat collection and analysis. Using a layer-by-layer fabrication process simplifies the creation of customizable 3D paper-based microfluidic devices. Polydimethylsiloxane (PDMS) plays a crucial role in fabricating these devices, serving as both a hydrophobic barrier that forms microchannels and an adhesive that bonds the layers. A flexible electrochemical sensing array is embedded within the 3D paper-based microfluidic network, allowing the device to monitor multiple substances in sweat simultaneously. The device can be worn on the skin for real-time sweat analysis, with signals transmitted wirelessly to the user through an integrated circuit board. This work presented a new method for fabricating 3D paper-based microfluidic electrochemical sensors, opening new opportunities for personalized diagnostics and physiological monitoring [101].

Chen et al. introduced a 3D-printed, flexible wearable health monitor fabricated via a novel one-step continuous manufacturing process, featuring self-supporting microfluidic channels and innovative single-atom catalyst-based bioassays for measuring sweat rate and concentrations of three biomarkers. The use of direct ink writing enables printing of a microfluidic device with self-supporting structures that harvest human sweat, thereby eliminating the need to remove sacrificial support materials and mitigating contamination and sweat evaporation issues associated with traditional sampling methods. Furthermore, employing a pick-and-place strategy in manufacturing enables precise integration of bioassays, enhancing production efficiency. A single-atom catalyst has been developed and incorporated into colorimetric bioassays to enhance sensitivity and accuracy. A feasibility study conducted on human skin successfully demonstrates the functionality and reliability of the health monitor, providing quantitative, in situ measurements of sweat rate and glucose, lactate, and uric acid concentrations during physical exercise [74].

Wu et al. proposed a wearable electrochemical sensor featuring gold nanoelectrode arrays fabricated on a nanoporous polycarbonate (PC) membrane, encapsulating lactate oxidase (LOx) within a chitosan (CS) hydrogel, designed to detect body temperature and sweat lactate concurrently. The flexible gold nanoporous electrodes not only increase the electrode surface area but also provide a nanoconfined environment that accelerates the catalytic reaction of LOx and helps regulate substrate concentration at the LOx surface, thereby reducing substrate inhibition. The sensor demonstrates a durability of up to 13 days and exhibits superior selectivity for sweat lactate detection over a broad linear range (0.01–35 mM) with a low detection limit (0.144 μM). Furthermore, temperature-dependent transmembrane currents passing through the sensor are utilized to estimate body temperature. Subsequently, multiple linear regression analysis was employed to account for the influence of temperature on lactate detection, successfully enabling the monitoring of lactate molecules in sweat and body temperature during exercise [102].

Asaduzzaman et al. proposed a functionalized hybrid nanoporous carbon (H-NPC)-encapsulated, flexible, three-dimensional porous graphene-based epidermal patch, which was first fabricated to monitor sweat glucose, lactate, pH, and temperature using simple, cost-effective, laser-engraved, and spray-coating techniques. The fabricated H-NPC-modified electrode markedly increased electrochemical surface area and electrocatalytic activity. Within the physiological sweat range (0–1.5 mM), the second-generation glucose sensor demonstrated exceptional sensitivity of 82.7 μA·mM^−1^·cm^−2^ with a limit of detection (LOD) of 0.025 μM. Furthermore, the lactate biosensor displayed an extraordinary linear response range (0–56 mM) owing to the incorporation of an outer diffusion-limiting layer (DLL) that regulates lactate flux reaching the enzyme, with comparable sensitivity and an LOD of 4 μM. Ultimately, an analytical correction approach involving pH and temperature adjustments was employed during in vivo testing. In addition to integrating various carbon-based materials with extensive metal–organic frameworks as transduction media, our research also advances the development of sensors that operate independently of pH and temperature corrections, thereby ensuring accurate measurements [103].

Padash et al. introduced a wearable microfluidic device designed for sweat analysis. The primary function of this device is to collect sweat from the skin and transport it to the sensor. It is fabricated using a 3D printer employing Multi-Jet Modelling (MJM) technology with flexible materials. The efficacy of the microfluidic device for electrochemical sensing of two distinct types of molecular analytes has been validated. Specifically, the electrochemical behavior of Paracetamol, serving as a model drug, and of ferro- and ferricyanide, as representative ionic analytes, was examined. The device’s performance was evaluated using both unmodified carbon and gold screen-printed electrodes. It is subsequently proposed for various potential analyses of sweat in both in vivo and in situ applications [17].

NajafiKhoshnoo et al. proposed, developed, and characterized an integrated, miniaturized, modular, wearable, battery-free, biocompatible, flexible, 3D-printed (WB2F3D) sensor system for on-demand, continuous, wireless, and real-time pH monitoring. The innovative use of 3D printing of nanomaterials on skin-like flexible substrates enables multimaterial, multilayer printing of sensors, reusable electronic/communication circuits, and antennas in a low-cost, time-efficient, and customizable manner. The battery-free, flexible readout system is designed to enable wireless, on-demand energy and data transmission for continuous, real-time pH monitoring. This sensor system exhibits high sensitivity (≈|51.76| mV pH^−1^), specificity, repeatability, and reproducibility across a pH range of 3.0–10.0, along with excellent mechanical flexibility and outstanding biocompatibility (cell viability ≥ 90%). It successfully demonstrates the ability to monitor pH changes in an ex situ hydrogel-based wound model. The WB2F3D sensor system aims to provide an integrated platform for accurate, on-demand, battery-free, wireless, and real-time human health monitoring, marking a step forward in personalized medicine [88].

Song et al. introduced a universal semisolid extrusion–based 3D printing technology to create an epifluidic elastic electronic skin (e3-skin) with high-performance multimodal physicochemical sensing capabilities. They show that the e3-skin can serve as a sustainable monitoring platform for tracking individuals’ real-time physiological states during daily activities. We also demonstrate that combining e3-skin data with machine learning enables us to predict an individual’s behavioral impairments (e.g., reaction time and inhibitory control) following alcohol intake. The e3-skin opens the way for future autonomous manufacturing of customizable wearable systems that may support widespread use for regular health monitoring and clinical applications [104].

Galliani et al. demonstrated the microfabrication of epidermal microfluidics within textiles using stereolithography (SLA) 3D printing. Flexible SLA resin creates impermeable, fluid-guiding microstructures in textile microfluidic modules. Their vertical stacking reduces device size and sample volume, while enabling on-body fluid collection, storage, and transport. Embedded internal modules serve as reservoirs and injection valves, releasing a specific volume of sweat to the sensing unit. The pressure gradient across the modules drives a vertically distributed, capillary-driven sweat flow, powered by the textile structure’s wicking ability. Fully integrated into garments, these systems allow a non-cumulative flow through an extended air-liquid interface, ensuring continuous sweat transfer and evaporation. For real-time sweat analysis. Galliani et al. also utilized a remotely screen-printed potassium (K^+^) ion detector. This modular method offers fabric-integrated, ergonomically designed microfluidics for multi-parameter detection via rapid additive manufacturing, thereby advancing point-of-care diagnostics [105]. Figure 5 shows a schematic of wearable sensor technologies for non-invasive sports monitoring through sweat analysis. Table 3 summarizes important studies on wearable microfluidic and electrochemical sensing systems for sweat-based physiological monitoring in sports.

Emerging materials and device strategies comprising graphene-based epidermal patches, nanoporous sensing layers, battery-free flexible sensors, and textile-integrated microfluidic systems are also presented, along with a case study by Ji et al. [99]. The diagram further elucidates the functional benefits of these systems, including modular and cost-efficient design, rapid customization, multiparameter sensing capability, and seamless integration with garments and smartphone-based data platforms. Typical sports applications are illustrated for endurance and high-intensity activities, emphasizing real-time assessment of hydration status, electrolyte equilibrium, fatigue, and metabolic process responses [74,99,100,101,102].

### 5.3. Wearable Sensors That Measure Molecular Biomarkers

In the study conducted by Cao et al., a three-dimensional paper-based microfluidic electrochemical integrated device (3D-PMED) was demonstrated for real-time monitoring of sweat metabolites. The 3D-PMED was fabricated by screen-printing wax patterns onto cellulose paper, which was subsequently folded four times to create five stacked layers: the sweat collector, vertical channel, transverse channel, electrode layer, and sweat evaporator. A sweat-monitoring device was realized by integrating a screen-printed glucose sensor onto a polyethylene terephthalate (PET) substrate with the fabricated 3D-PMED. The sweat flow process within the 3D-PMED was modeled using red ink to illustrate its capacity to collect, analyze, and evaporate sweat, leveraging the capillary action of filter paper and the hydrophobicity of wax. The glucose sensor was engineered with high sensitivity (35.7 μA mM^−1^ cm^−2^) and a low detection limit (5 μM) to account for the low glucose concentration in sweat. An on-body experiment was conducted to validate the practicality of the three-dimensional sweat monitoring device. This 3D-PMED can be readily expanded to simultaneously monitor alternative sweat electrolytes and metabolites [107].

Nesaei et al. introduced a 3D-printed flexible electrochemical biosensor for glucose detection. We demonstrate that our biosensor exhibits a linear response over a glucose concentration range of 100–1000 μM. The sensitivity of the glucose biosensor is estimated at 17.5 nA μM^−1^, and the calculated detection limit (S/N = 3) is 6.9 μM. The electrochemical performance and surface properties of the printed sensors highlight the promising advantages of this technique over conventional screen printing. These benefits include higher sensitivity and specificity, as well as lower material consumption [108].

Katseli et al. introduced a wearable glucose-monitoring device shaped like an electrochemical ring (e-ring), made using 3D printing. The 3D-printed e-ring features three carbon-based plastic electrodes, created with a conductive filament, embedded inside a flexible, ring-shaped plastic holder made from a nonconductive filament. The e-ring is coated with a gold film through electrodeposition and connected to a small potentiostat that can be controlled directly via a smartphone. This setup allows for nonenzymatic amperometric self-testing of glucose levels in human sweat. Optical and electrochemical methods are used to characterize the e-ring. The device is durable against mechanical bending and enables noninvasive glucose detection in sweat within the physiologically relevant range of 12.5–400 μmol L^−1^, free from interference by common electroactive metabolites. The 3D-printed e-ring helps bridge the gap between current fabrication and sensing technologies and the operational features needed for glucose self-monitoring, serving as a model for in-house-developed wearable sensors [109].

Singh et al. engineered an aptamer with a pseudoknot that restricts it to two states, thereby reducing background signals and enabling high sensitivity. An electrochemical pH sensor provides pH-compensated amperometric measurements. Device operation was demonstrated in vitro with a broad linear dynamic range (1 pM–1 μM), covering the physiological range, and a sub-picomolar detection limit (0.2 pM) in sweat. Real-time, on-body measurements were taken from human subjects using an induced-stress protocol, showing in situ signal regeneration and the ability to continuously monitor dynamic cortisol fluctuations for up to 90 min. The reported device could improve prognosis and support personalized treatments [110].

Chakoma et al. developed a passive, wearable sensing system that uses a molecularly imprinted polymer and radio-frequency technology (MIP-RF) to assess sweat cortisol levels in real time without invasive procedures. The system is wireless, flexible, reuses power from a battery, is environmentally stable, and suitable for long-term use, employing an inductance-capacitance transducer. This transducer detects cortisol concentrations by measuring shifts in resonant frequency with high sensitivity (approximately 160 kHz per log(µM)) across the physiological range of 0.025–1 µM. It includes near-field communication (NFC) for wireless, battery-free operation and a 3D-printed microfluidic channel for collecting sweat directly on-site, allowing daily cortisol monitoring. Validation through morning and evening measurements confirms its ability to track circadian variations in sweat cortisol. A 28-day stability test and low-cost 3D printing of nanomaterials improve its affordability and reusability. This breakthrough opens the door to convenient, on-demand health monitoring outside laboratories, using wearable technology to detect molecular stress biomarkers [111].

Nah et al. created a wearable electrochemical immunosensor with a microfluidic channel, based on a Ti_3_C_2_T_x_ MXene-loaded laser-burned graphene (LBG) 3D electrode for noninvasive cortisol detection in sweat. Using PDMS as a flexible substrate, LBG was transferred onto it after removing the polyamide film. Laser line gaps caused disconnection between graphene flakes, reducing performance, but coating with conductive Ti_3_C_2_T_x_ MXene addressed this. The system was made via 3D printing and PDMS, attached to the skin to collect sweat that flows through the channel to the chamber. MXene loading was confirmed by FESEM and XPS. Optimized, the sensor showed linear detection over 0.01–100 nM and a sensitivity of 88 pM, demonstrating its suitability for point-of-care cortisol detection [112].

Weng et al. developed a portable 3D microfluidic origami biosensor that uses a smartphone to detect cortisol in human sweat. The device employs MoS2 nanosheets and fluorescent aptamers, and features a multilayered chip designed for low-volume sweat collection, transport, and analysis. The design parameters, including paper substrates, MoS2 concentration, and incubation period, were optimized to encompass a physiological range of 10–1000 ng/mL. Under optimal conditions, it detected cortisol with a limit of detection (LOD) of 6.76 ng/mL at 3σ in artificial sweat within 25 min and exhibited high selectivity. Its performance was comparable to that of an enzyme-linked immunosorbent assay (ELISA), with a correlation coefficient of 0.988, and it yielded reliable results with authentic sweat samples. This portable, rapid, economical, and non-invasive biosensor offers a convenient point-of-care solution for cortisol monitoring [1].

**Figure 6:**
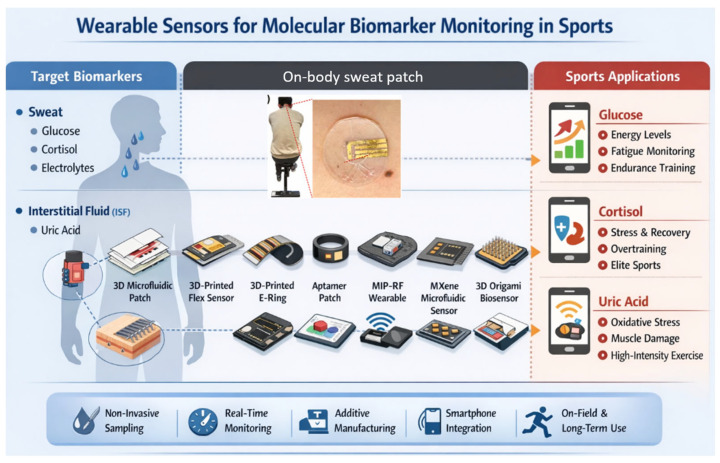
The overview of wearable sensing platforms for molecular biomarker monitoring in sports highlights key target biomarkers detected from non-invasive biofluids, mainly sweat (glucose, cortisol, and electrolytes) and interstitial fluid (uric acid), and their importance to physiological status during exercise. Image generated by AI using ChatGPT (GPT-5, OpenAI, San Francisco, CA, USA).

Parrila et al. introduced an economical and adaptable wearable patch that uses 3D-printed microneedle arrays to enhance electrochemical sensors. The design features a plug-in configuration with a three-electrode setup, facilitating quick deployment as a voltammetric sensor. Sputtering technology is used to produce microneedle-array electrodes composed of nanostructured gold. This material demonstrates exceptional electrochemical performance owing to its nanostructured surface and offers antibiofouling properties against proteins. The MES’s ability to penetrate porcine skin without causing damage has been successfully demonstrated. Subsequently, the analytical characterization of uric acid (UA) was completed in buffer solution, protein-enriched solution, simulated interstitial fluid (ISF), and an ex vivo setup, exhibiting notable performance within the physiological UA range and excellent reversibility, with no difference between upward and downward calibrations during skin piercing. Finally, an initial on-body evaluation confirms the MES’s ability to penetrate the skin, thereby validating the wearable patch’s robustness for in vivo analysis. The on-body test showed a 5.8% difference in signal between pre- and post-application measurements. These findings underscore the potential of a versatile microneedle-based electrochemical cell employing nanostructured gold, enabling the rapid development of innovative sensors for interstitial fluid monitoring [3]. Figure 6 illustrates wearable sensing platforms for monitoring molecular biomarkers in sports. Table 4 summarizes studies on wearable electrochemical and microfluidic sensor systems used for monitoring molecular biomarkers in sports settings.

Depiction of on-body sweat patch application to the lower neck of the subjects during trials. Several wearable sensing technologies are shown, including microfluidic patches, flexible and 3D-printed sensors, aptamer-based platforms, molecularly imprinted polymer (MIP)-enabled radiofrequency wearables, MXene-based microfluidic sensors, and origami-inspired biosensors and on-body sweat patch application to the lower neck of the subjects during trials [110]. Figure 6 also emphasizes how biomarker data can be applied practically in sports, such as tracking energy use, stress and recovery, fatigue, oxidative stress, and muscle damage. Key features of these systems—non-invasive sampling, real-time monitoring, additive manufacturing, smartphone integration, and suitability for on-field and long-term use are also summarized.

### 5.4. Cardiovascular Monitoring in Sports Applications

Cardiovascular monitoring has become an indispensable component of modern sports science because it provides continuous information regarding exercise intensity, physiological adaptation, recovery status, and potential cardiovascular abnormalities. Traditional laboratory-based cardiovascular assessments are increasingly being replaced by wearable and textile-integrated systems that enable real-time monitoring during training and competition. Recent advances in wearable biosensors have expanded the scope of cardiovascular monitoring beyond heart rate measurements to include respiratory rate, pulse wave characteristics, blood oxygen saturation, blood pressure-related indices, and cardiovascular risk assessment [21,22,23,113,114,115,116,117].

Heart rate remains the most widely monitored cardiovascular parameter in sports because of its strong relationship with exercise intensity and training load. However, recent studies have highlighted the limitations of conventional wearable devices during dynamic exercise. Van Oost et al. [116] evaluated the performance of wearable devices under varying physiological conditions and reported that measurement accuracy can be substantially affected by movement artifacts and rapid cardiovascular fluctuations. Their findings demonstrated that chest-based monitoring systems generally provide higher reliability than wrist-worn devices, particularly during transitions between exercise intensities. These observations are especially relevant for athletes participating in high-intensity interval training and endurance sports, where rapid heart rate changes frequently occur.

In addition to heart rate monitoring, respiratory monitoring has emerged as an important complementary parameter for evaluating cardiovascular performance and exercise adaptation. Kobayashi et al. [117] developed a low-compression smart garment incorporating a double-layer capacitive bending-angle sensor capable of continuously monitoring respiratory activity. The system demonstrated excellent agreement with conventional spirometry measurements, highlighting the feasibility of comfortable long-term respiratory monitoring in athletic environments. Since respiratory rate is closely associated with ventilatory threshold, aerobic capacity, and cardiovascular strain, integrating respiratory sensing with cardiovascular monitoring may significantly improve athlete assessment.

Textile-integrated wearable systems have received considerable attention because they provide a more natural and comfortable solution than conventional electrodes. Al-Azzawi et al. [23] introduced a three-dimensional knitted smart T-shirt capable of simultaneously monitoring heart rate and respiratory rate. The textile-based platform showed strong agreement with commercial monitoring devices while offering enhanced wearability and reduced discomfort during prolonged use. Such smart garments represent an important advancement for sports applications because they enable unobtrusive physiological monitoring without restricting athlete movement.

Recent developments in cardiovascular wearable technologies have expanded toward multimodal sensing approaches. Xie et al. [114] reviewed emerging wearable cardiovascular monitoring systems and emphasized the growing integration of electrocardiography (ECG), photoplethysmography (PPG), bioimpedance sensing, flexible pressure sensors, and wireless communication technologies. The authors highlighted that multimodal systems provide a more comprehensive representation of cardiovascular status than single-sensor platforms by simultaneously monitoring multiple physiological signals. Such integration is particularly advantageous in sports settings where physiological responses are influenced by numerous internal and external factors.

The importance of advanced cardiovascular monitoring extends beyond performance optimization and increasingly contributes to athlete safety. The expert consensus statement published by Gajda et al. [22] emphasized the role of wearable heart rate monitors in the detection of exercise-induced arrhythmias and cardiovascular abnormalities. The authors noted that future sports monitoring systems should incorporate improved artifact resistance, ECG-based monitoring capabilities, reliable wireless transmission, and user-friendly designs to facilitate both training management and cardiovascular risk assessment. These recommendations reflect the growing recognition that wearable devices may serve as valuable tools for the early identification of potentially life-threatening cardiac events in athletes.

Photoplethysmography-based wearable devices continue to dominate commercial sports monitoring applications because of their low cost and ease of integration. However, recent evaluations indicate that PPG measurements remain susceptible to motion artifacts, changes in skin contact pressure, sweating, and peripheral blood flow variations [21,115]. Consequently, several studies have proposed combining PPG with complementary sensing technologies such as bioimpedance, ECG, and artificial intelligence-assisted signal processing to improve measurement accuracy during vigorous physical activity [23,113].

Artificial intelligence is emerging as a transformative technology in sports cardiovascular monitoring. Sadeghi et al. [115] demonstrated the integration of AI algorithms with wearable physiological monitoring platforms to enable continuous assessment of vital signs and automated identification of abnormal physiological patterns. Machine learning-assisted systems may enhance the interpretation of cardiovascular signals by distinguishing genuine physiological responses from motion-related artifacts, thereby improving the reliability of real-time athlete monitoring. Such approaches are expected to play a critical role in future personalized training and health management strategies.

From a clinical perspective, cardiovascular monitoring is becoming increasingly important for athlete screening and prevention of sudden cardiac events. The scientific statement published in the Journal of the American College of Cardiology emphasized that wearable cardiovascular technologies have significant potential for early detection of arrhythmias, monitoring cardiovascular adaptation to exercise, and facilitating individualized exercise prescription [116,118]. As shown in Table 5, the report further highlighted the necessity for rigorous validation studies before wearable cardiovascular devices can be fully integrated into clinical decision-making and athlete management programs.

Overall, recent advances indicate that cardiovascular monitoring in sports is evolving from simple heart rate tracking toward intelligent, multimodal, and textile-integrated sensing platforms. The convergence of ECG, PPG, respiratory monitoring, bioimpedance sensing, artificial intelligence, and smart textile technologies offers unprecedented opportunities for continuous cardiovascular assessment in real-world sports environments. Nevertheless, challenges including motion artifacts, lack of standardization, limited validation during high-intensity exercise, and insufficient long-term clinical evidence remain significant barriers to widespread adoption. Future research should focus on developing multimodal wearable systems that simultaneously monitor cardiovascular, respiratory, and biomechanical parameters while maintaining high accuracy, comfort, and long-term stability during athletic performance.

## 6. Technical Challenges and Translational Hurdles

### 6.1. Material Incompatibility and Multi-Material Printing Constraints

#### 6.1.1. Physical and Electrophysiological Sensors

Wearable sensors designed to measure physical and electrophysiological parameters rely heavily on the integration of highly conductive nanomaterials (such as Ti_3_C_2_T_x_ MXene, graphene, and PEDOT:PSS) with mechanically flexible substrates, including elastomers, hydrogels, and textiles. Studies by Zhang et al. (MPCA) [91], Lu et al. [92], Lee et al. [93], and Garg et al. [94] show that MXene-based conductive networks significantly improve sensitivity and signal stability; however, combining these materials with soft matrices presents significant manufacturing challenges. For example, Lu et al. [92] achieved remarkable stretchability (up to 2400%) using MXene-reinforced double-network hydrogels, but this required carefully staged fabrication rather than simple multi-material 3D printing. Conversely, Zhang et al. (AMSS) [95] used PLA-based 4D printing for programmable strain sensing, but PLA’s thermal processing window limits its compatibility with conductive nanomaterials, which often require post-treatment or low-temperature deposition. Textile-based systems such as those by Lee et al. [93] and Driscoll et al. [98] (MXtrodes) further highlight compatibility issues: while textile substrates improve comfort and wearability, establishing durable electrical interfaces between fibers and nanomaterial coatings remains challenging, especially under the repeated deformation common in sports. No current platform enables single-step, scalable printing of conductive nanomaterials and highly deformable substrates without compromising electrical or mechanical integrity.

#### 6.1.2. Chemical Sensors

Chemical wearable sensors require the integration of electrodes, selective membranes, enzymes, and microfluidic architectures, thereby significantly increasing the risk of material incompatibility. In modular systems such as the “Lego Bricks” sensor by Ji et al., this issue is reduced by separating functional layers; however, this modularity increases assembly complexity. Similarly, Kim et al. [100] and Chen et al. [74] used 3D printing to integrate microfluidics and sensing elements, yet enzymatic layers (e.g., LOx in Wu et al. [102], glucose oxidase in Asaduzzaman et al. [103]) could not be printed simultaneously with structural components because enzymes are sensitive to heat, solvents, and shear forces. Compared to physical sensors, chemical sensors face greater material incompatibility because biochemical recognition layers often require post-processing steps, which limit true monolithic fabrication.

#### 6.1.3. Molecular Biomarker Sensors

Material incompatibility is most significant in molecular biomarker sensors. Aptamers (Singh et al. [110], Weng et al. [1]), antibodies (Nah et al. [112]), and MIPs (Chakoma et al. [111]) are inherently incompatible with standard 3D/4D printing conditions. For instance, Singh et al. [110] achieved sub-picomolar cortisol detection using a pseudoknot-assisted aptamer; however, the sensing layer had to be fabricated separately and subsequently integrated. Likewise, Nah et al. [112] enhanced signal transduction by coating laser-burned graphene with MXene; however, this hybrid approach required multiple fabrication steps.

While physical sensors struggle mainly with mechanical–electrical compatibility, molecular sensors face chemical and biological incompatibility, making translation to scalable manufacturing significantly more challenging.

### 6.2. Response Time Lag and Dynamic Mismatch

#### 6.2.1. Physical and Electrophysiological Sensors

Physical and electrophysiological signals (strain, pressure, EMG) occur on millisecond timescales. Most MXene-based strain and EMG sensors (Lu et al. [92], Garg et al. [94]) successfully meet these temporal demands. However, 4D-printed systems, such as those by Zhang (AMSS) [95] and Deng et al. [96], introduce time-dependent shape transformations governed by thermal or phase-change mechanisms. While these systems provide adaptive joint conformity, their actuation times (seconds to minutes) lag behind the rapid biomechanical events in sports, limiting their use to quasi-static adaptation rather than real-time correction.

#### 6.2.2. Chemical Sensors

Chemical sensing is subject to intrinsic delays due to sweat secretion, transport, and reaction kinetics. Microfluidic designs by Hashimoto et al. [83], Islam et al., and Galliani et al. reduce transport delays but cannot eliminate them completely. Enzymatic sensors (Wu et al. [102], Asaduzzaman et al. [103]) also face reaction-limited response times. While correction algorithms can account for temperature and pH effects, they do not eliminate latency, which remains a problem for real-time decision-making in high-intensity sports.

#### 6.2.3. Molecular Biomarker Sensors

Molecular biomarker detection naturally exhibits slower dynamics due to binding kinetics. Singh et al. [110] have partially addressed this issue through aptamer engineering, which enables real-time cortisol monitoring for 90 min; however, achieving sub-second temporal resolution remains beyond current capabilities. Compared with chemical analytes such as lactate or glucose, cortisol and cytokines exhibit more gradual physiological fluctuations, which somewhat alleviates this limitation. Nonetheless, response lag remains a critical barrier for acute stress monitoring during competition.

### 6.3. Sterilization, Biocompatibility, and Long-Term Wear

#### 6.3.1. Physical and Electrophysiological Sensors

Dry electrodes, such as MXtrodes (Garg et al. [94], Driscoll et al. [98]), exhibit superior skin compatibility compared to gel-based electromyography (EMG) electrodes. Nevertheless, extended exposure times raise concerns about nanomaterial leaching and mechanical irritation, particularly in environments with high sweat levels.

#### 6.3.2. Chemical Sensors

Chemical sensors face dual biocompatibility challenges: polymeric substrates and biochemical reagents. Enzyme degradation during sterilization requires mild, often inadequate, sterilization protocols. Studies such as NajafiKhoshnoo et al. [88]. demonstrate progress through battery-free, biocompatible designs, yet long-term epidermal studies remain limited.

#### 6.3.3. Molecular Biomarker Sensors

Molecular sensors exhibit the highest sensitivity to sterilization processes. Aptamers and antibodies are prone to denaturation, whereas MIP-based systems Chakoma et al. [111] provide enhanced stability but demonstrate decreased selectivity. Long-term dermatological safety data concerning these systems within athletic populations are presently unavailable.

Despite significant technological advances, several barriers continue to hinder the large-scale clinical translation of wearable biosensors. Regulatory approval requires comprehensive analytical validation, biocompatibility assessment, electrical safety evaluation, and demonstration of clinical performance under real-world conditions. Compliance with standards such as ISO 10993 and relevant IEC guidelines is essential, yet many emerging wearable systems remain at the laboratory validation stage. Calibration represents another critical challenge because sensor responses are influenced by environmental conditions, biofluid composition, sensor aging, and individual physiological characteristics. Consequently, universal calibration models often fail to provide consistent performance across diverse user populations. Recent approaches have therefore focused on personalized calibration strategies, machine learning-assisted correction algorithms, and multimodal data integration to improve measurement accuracy. Standardization also remains limited within the wearable biosensing field. Variations in sampling protocols, sensor placement, testing environments, and data-processing methodologies frequently complicate direct comparisons among studies and devices. The development of harmonized validation procedures and standardized reference materials will be essential for accelerating regulatory acceptance and commercial adoption. Furthermore, substantial inter-individual variability exists in sweat secretion, analyte partitioning, skin physiology, hydration status, age, sex, and metabolic activity. These factors can significantly affect the relationship between measured biofluid concentrations and underlying physiological conditions. Future wearable systems will therefore require adaptive sensing strategies, personalized calibration frameworks, and advanced computational models that account for user-specific physiological differences while maintaining clinical reliability.

## 7. Future Outlook: Toward a Digital Skin Ecosystem

### 7.1. Beyond 4D Printing: Toward Information-Embedded (5D) Systems

For physical sensors, integrating signal processing directly into printed architectures could enable on-device interpretation of strain or EMG patterns, reducing latency and energy consumption. In the realm of chemical sensing, information-embedded printing may enable adaptive sampling strategies, as illustrated conceptually by machine learning-assisted platforms such as the e3-skin developed by Song et al. [104] for molecular biomarker sensors; printed logic may regulate sensing events based on physiological thresholds, thereby addressing response delays and reducing false positives.

### 7.2. Integration with Soft Robotics and Closed-Loop Feedback

Physical sensors are the closest to realizing closed-loop systems. Strain sensors integrated with soft actuators may offer real-time posture correction, building upon the concepts demonstrated by Cheng et al. in orthotic devices. Chemical sensors may provide insights into hydration or fatigue levels through haptic feedback, whereas molecular sensors could facilitate stress-responsive interventions. Nevertheless, complete integration remains predominantly theoretical, especially concerning molecular biomarkers.

### 7.3. Future Challenges and Opportunities in Quantitative Sweat and ISF Biosensing

Despite advances in wearable biosensors, matching non-invasive measurements to blood diagnostics remains challenging. A key issue is the delay between blood analyte changes and their appearance in sweat or interstitial fluid, especially for rapidly fluctuating biomarkers like glucose, lactate, cortisol, and stress hormones, which may not reflect blood changes immediately. Another challenge is the low concentration of many biomarkers in sweat, such as cortisol, testosterone, estradiol, and inflammatory mediators, requiring highly sensitive sensors that may detect low levels amid complex matrices. Variability between individuals, including differences in sweat rate, gland density, skin permeability, hydration, and metabolic activity, complicates data interpretation. Variations in dermal transport also affect the correlation between ISF and blood analyte levels, often resulting in poor agreement with blood-based reference values.

Future wearable biosensors must therefore move beyond simple analyte detection and toward quantitative physiological interpretation. Smart materials such as adaptive hydrogels, osmotic interfaces, self-regulating microfluidics, and Janus biofluid-harvesting structures may improve the consistency of sample collection and reduce temporal delays associated with biofluid transport. Simultaneously, nanostructured transducers based on MXenes, graphene derivatives, metallic nanostructures, and conductive polymer composites may provide the sensitivity required for reliable detection of ultra-low-abundance biomarkers.

The integration of these advanced materials with artificial intelligence-assisted signal processing, personalized calibration algorithms, multimodal sensing strategies, and digital-twin-based physiological modeling may further mitigate inter-individual variability and differences in analyte partitioning. Such approaches may enable dynamic correction of sweat–blood and ISF–blood relationships, ultimately improving both temporal and absolute correlation with gold-standard clinical measurements. Future progress in wearable biosensing may therefore depend not only on advances in sensing materials but also on the development of intelligent biointerfaces capable of accurately translating non-invasive biofluid measurements into clinically actionable physiological information.

## 8. Comparative Analysis and Emerging Trends in Wearable Sports Biosensors

Recent advances in wearable sports biosensors have been driven by the convergence of nanomaterials, flexible electronics, additive manufacturing, and intelligent material systems. Across the reviewed studies, MXene-based materials, conductive hydrogels, graphene derivatives, and metallic nanostructures emerged as some of the most promising sensing platforms due to their high sensitivity, mechanical flexibility, and compatibility with wearable applications [26,27,28,29]. Among these materials, MXene-based systems demonstrated particularly strong performance in electrophysiological monitoring and strain sensing owing to their excellent electrical conductivity, low skin–electrode impedance, and mechanical robustness [29,119].

From a manufacturing perspective, 3D printing has enabled personalized sensor fabrication, anatomical customization, and integration of complex microfluidic architectures for sweat management [75,76,77]. However, most 3D-printed wearable sensors remain passive systems that rely on fixed structural designs. In contrast, 4D printing introduces stimuli-responsive functionality through shape-memory polymers, self-healing materials, and adaptive hydrogels, allowing wearable devices to dynamically respond to environmental and physiological changes [75,76,77]. These adaptive capabilities offer significant advantages for maintaining sensor–skin conformity, signal stability, and long-term operational reliability during intensive physical activity [75,76,77].

Despite substantial technological progress, several challenges continue to limit large-scale clinical and commercial implementation. Material durability under prolonged mechanical stress, long-term biocompatibility, manufacturing reproducibility, device calibration, and regulatory approval remain critical barriers [26,27,28,29,75,76,77]. In addition, many reported systems have been validated only under controlled laboratory conditions, while large-scale human studies remain limited [75,76,77].

Emerging trends indicate a transition toward fully integrated multimodal platforms capable of simultaneously monitoring mechanical, electrophysiological, biochemical, and molecular biomarkers. Future wearable sports biosensors are expected to integrate advanced nanomaterials, intelligent 4D-printed structures, artificial intelligence-assisted data analysis, and wireless health-monitoring networks. Such developments may facilitate the creation of adaptive, self-regulating sensing systems capable of providing continuous and personalized physiological assessment in real-world athletic environments.

## 9. Conclusions

Adaptive materials and 4D printing technologies have the potential to enhance the physical interaction between tissue and device by increasing the mechanical adaptability of wearable biosensors. These technologies can improve signal stability and reduce performance degradation during long-term use through programmable structures that can respond to deformation. In particular, the integration of nanomaterial-based high-precision transduction mechanisms with intelligent structural adaptation capabilities is seen as an important approach to improve measurement accuracy and reliability in constantly changing physiological conditions and environments. However, fundamental translational barriers still prevent these systems from becoming widely used in clinical practice. Issues like lack of clinical validation, technical challenges in scaling up manufacturing, and insufficient long-term biocompatibility data currently prevent their routine clinical application. Therefore, future research should aim to systematically assess how programmable materials affect real-world device performance, develop strategies for multi-parameter sensor integration, and create standardized protocols for clinical testing. In this context, adaptive wearable platforms represent a major shift from systems that only passively monitor biophysiological data to intelligent epidermal interfaces capable of actively engaging with users. This change signifies a crucial step toward next-generation biomedical devices that enable personalized health monitoring, real-time biofeedback, and dynamic therapeutic interventions.

## Figures and Tables

**Table 3:** Overview of key studies on wearable microfluidic and electrochemical sensing systems for sweat-based physiological monitoring in sports.

Study	Target Analytes/Parameters	Fabrication & System	Sports Relevance	Relative Advantages	Current Limitations	References
Ji et al.	Na^+^, K^+^	Screen + wax printing, modular paper/PET system	Endurance sports, prolonged exercise, and hydration monitoring	Modular and scalable architecture; low-cost manufacturing; suitable for multiplex sweat analysis	Limited integration of advanced data analytics and multimodal sensing	[99]
Kim et al.	Multiple electrolytes	3D-printed AIIW patch with microfluidics	Personalized training, endurance, and team sports	Highly customizable 3D-printed design; multiplex biomarker monitoring	Dependence on fabrication precision and user-specific fitting	[100]
Mei et al.	Multiple sweat analytes	3D paper-based microfluidic electrochemical sensor	General fitness, endurance exercise	Simple fabrication process and wireless real-time monitoring capability	Limited long-term validation under intensive sports conditions	[101]
Chen et al.	Sweat rate, glucose, lactate, and uric acid	3D-printed microfluidics with single-atom catalysts	High-intensity training, metabolic monitoring	High analytical sensitivity with quantitative in situ biochemical analysis	Increased system complexity due to catalyst integration and microfluidics	[74]
Wu et al.	Lactate, temperature	Nanoporous gold electrodes with LOx	High-intensity and interval sports	Accurate lactate sensing with temperature compensation; broad analytical range	Focused primarily on lactate monitoring rather than multimodal assessment	[102]
Asaduzzaman et al.	Glucose, lactate, pH, temperature	3D graphene-based epidermal patch	Fatigue and metabolic stress monitoring	Multimodal physiological monitoring with signal-correction algorithms	Complex system integration and higher power/data-processing requirements	[106]
Padash et al.	Model drugs, ionic analytes	3D-printed microfluidic device	Proof-of-concept for sports sweat analysis	Flexible microfluidic architecture demonstrating versatile transport behavior	Primarily proof-of-concept with limited physiological validation	[17]
NajafiKhoshnoo et al.	pH	Battery-free 3D-printed wireless system	Endurance sports, prolonged exertion	Battery-free operation and wireless communication; high user comfort	Restricted to a limited number of analytes and sensing functions	[88]
Song et al.	Multimodal physicochemical signals	3D-printed epifluidic electronic skin	Performance and cognitive state monitoring	Integration of multimodal sensing with machine learning-assisted analysis	Computational complexity and limited large-scale validation studies	[104]
Galliani et al.	K^+^ (demonstrated)	Textile-integrated microfluidics via SLA	On-garment monitoring for endurance sports	Direct textile integration enabling continuous and unobtrusive monitoring	Limited analyte range demonstrated and potential textile durability concerns	[105]

**Table 4:** A comparative overview of studies on wearable electrochemical and microfluidic sensor systems used for monitoring molecular biomarkers in sports settings.

Study	Target Biomarker(s)	Sensing/Fabrication Approach	Sports Relevance	Relative Advantages	Current Limitations	References
Cao et al.	Glucose (expandable to others)	Paper-based 3D microfluidic electrochemical device	Endurance sports, metabolic monitoring	Low-cost fabrication, simple operation, and effective sweat handling through 3D microfluidics	Limited biomarker panel and proof-of-concept stage validation	[107]
Nesaei et al.	Glucose	3D-printed flexible electrochemical biosensor	Training load and energy balance	Enhanced sensitivity with reduced material consumption and flexible architecture	Primarily focused on glucose monitoring; broader applicability remains to be demonstrated	[108]
Katseli et al.	Glucose	3D-printed electrochemical ring (nonenzymatic)	Field sports, continuous self-monitoring	Nonenzymatic detection, smartphone integration, and high mechanical robustness	Limited clinical validation under real-world sports conditions	[109]
Singh et al.	Cortisol (pH-compensated)	Aptamer-based electrochemical patch	Stress, overtraining, and recovery assessment	Ultra-low detection limit, high selectivity, and real-time sensor regeneration	Aptamer stability and long-term operational performance require further investigation	[110]
Chakoma et al.	Cortisol	MIP-RF wearable with NFC	Long-term stress monitoring in athletes	Battery-free operation, NFC, and reusable sensing platform	Limited multiplexing capability and dependence on MIP fabrication quality	[111]
Nah et al.	Cortisol	MXene–LBG microfluidic immunosensor	Acute and chronic stress during training	High sensitivity through MXene-enhanced conductivity and integrated microfluidics	Potential long-term MXene oxidation and fabrication complexity	[112]
Weng et al.	Cortisol	3D microfluidic origami + smartphone readout	On-field stress screening	Portable smartphone-assisted analysis with ELISA-comparable performance	Requires additional sample-processing steps compared with fully integrated wearable systems	[1]
Parrilla et al.	Uric acid (ISF)	3D-printed microneedle electrochemical patch	High-intensity and endurance sports	Direct access to interstitial fluid, reduced biofouling, and improved analytical reliability	Microneedle-based systems may face user acceptance and regulatory challenges	[3]

**Table 5:** Comparative Analysis of Cardiovascular Monitoring Technologies for Sports Applications.

Study	Target Biomarker(s)	Sensing/Fabrication Approach	Sports Relevance	Key Advantage	References
Gajda et al.	Heart rate, arrhythmia-related signals	Wearable heart rate monitoring systems and ECG-based platforms	Athlete safety, arrhythmia detection, cardiovascular risk assessment	Established recommendations for next-generation sports cardiovascular monitoring systems	[22]
Al-Azzawi et al.	Heart rate, respiratory rate	3D knitted smart textile T-shirt with integrated textile sensors	Continuous physiological monitoring during sports activities	High wearability and simultaneous HR–RR monitoring without restricting movement	[23]
Xie et al.	HR, ECG, PPG, blood pressure-related signals, vascular parameters	Multimodal wearable cardiovascular sensors integrating ECG, PPG, bioimpedance, flexible electronics, and wireless communication	Comprehensive cardiovascular assessment during exercise	Simultaneous acquisition of multiple cardiovascular parameters	[114]
Sadeghi et al.	Heart rate, SpO_2_, body temperature, vital signs	AI-assisted wearable physiological monitoring platform	Real-time athlete monitoring and abnormal physiological event detection	Machine learning improves interpretation and reduces false alarms	[115]
Van Oost et al.	Heart rate (HR)	Wearable ECG and PPG-based commercial devices	Exercise intensity monitoring, training load assessment	Demonstrated superior accuracy of chest-based systems during dynamic exercise compared to wrist-worn devices	[116]
Kobayashi et al.	Respiratory rate	Low-compression smart garment incorporating double-layer capacitive bending-angle sensors	Monitoring ventilatory response, aerobic performance, and recovery	Excellent agreement with spirometry while maintaining athlete comfort	[117]

## Data Availability

No new data were created or analyzed in this study. Data sharing is not applicable to this article.
